# Mammalian expression vectors for metabolic biotinylation tandem affinity tagging by co-expression in *cis* of a mammalian codon-optimized BirA biotin ligase

**DOI:** 10.1186/s13104-018-3500-9

**Published:** 2018-06-14

**Authors:** Marina Ioannou, Dimitris N. Papageorgiou, Vasily Ogryzko, John Strouboulis

**Affiliations:** 1 Institute of Molecular Biology and Biotechnology, Foundation of Research & Technology Hellas, 100 Nikolaou Plastira Street, 70013 Heraklion, Crete Greece; 2 Division of Molecular Oncology, Biomedical Sciences Research Center “Alexander Fleming”, 34 Fleming Street, 166 72 Vari, Greece; 3 UMR8126, Université Paris-Sud 11, CNRS, Institut de Cancérologie Gustave Roussy, 94805 Villejuif, France; 4 Present Address: Division of Proteomics Stem Cells and Cancer, German Cancer Research Center (DKFZ), Im Neuenheimer Feld 581, 69120 Heidelberg, Germany

**Keywords:** Expression vectors, Tandem affinity purification, Metabolic biotinylation tagging, Avi tag, FLAG tag, BirA biotin ligase

## Abstract

**Οbjective:**

To construct mammalian expression vectors for the N- or C-terminal tagging of proteins with a tandem affinity tag comprised of the biotinylatable Avi tag and of a triple FLAG tag.

**Results:**

We constructed and tested by transient transfections mammalian expression vectors for the co-expression from a single plasmid of N- or C-terminally tagged proteins bearing a tandem affinity tag comprised of the biotinylatable Avi tag and of a triple FLAG tag separated by a tobacco etch virus (TEV) protease cleavage site, together with a mammalian codon-optimized BirA biotin ligase fused to green fluorescent protein. We also describe platform vectors for the N- or C-terminal AVI-TEV-FLAG tagging of any complementary DNA of choice. These vectors offer versatility and efficiency in the application of metabolic biotinylation tandem affinity tagging of nuclear proteins in mammalian cells.

**Electronic supplementary material:**

The online version of this article (10.1186/s13104-018-3500-9) contains supplementary material, which is available to authorized users.

## Introduction

Metabolic biotinylation tagging of proteins offers a high affinity tagging approach with an increasing number of applications in mammalian cells [1]. It involves the co-expression in cells of the *E. coli* BirA biotin ligase together with the protein of interest fused to a small artificial peptide tag (the Avi tag) which is specifically recognized and efficiently biotinylated by BirA in cells [2, 3]. Biotin-tagged proteins can be bound very tightly by avidin and streptavidin (dissociation constant *K*d = 10^−15^), a fact that has been widely exploited in many affinity-based biochemical applications [4]. Furthermore, biotinylation tagging offers a number of advantages for the purposes of protein tagging. First, there are only five, mostly mitochondrial, naturally biotinylated proteins ensuring low nonspecific background [5]. Second, high stringencies can be employed in any biotin/(strept)avidin affinity purification or detection protocol, without fear of losing the tagged protein. Third, a great variety of biotin/(strept)avidin-related reagents are commercially available for protein applications. Lastly, protein biotin tagging can be further extended by combining combination with other epitope tags, fused in tandem to the protein of interest [6, 7].

However, as versatile as biotinylation tagging may be, it is somewhat complicated by the fact that it is a binary system relying on the simultaneous expression of the Avi-tagged protein of interest and of the BirA protein biotin ligase. In addition, expression of the prokaryotic BirA biotin ligase in mammalian cells can be problematic due to inefficient translation as a result of differences in codon usage between bacterial and mammalian cells [8]. In order to overcome these challenges, we describe here the construction of mammalian expression vectors for the expression of tagged proteins bearing an N- or C-terminal Avi-triple FLAG tandem affinity tag and, concurrently, of a mammalian codon-optimized (“humanized”) BirA-GFP fusion. The N- or C-terminally tagged protein and hBirA-GFP are driven by two separate promoters on the same plasmid and can be used for transient or stable transfections in mammalian cells.

## Main text

### Methods

#### Plasmid constructs

**Fig. 1:**
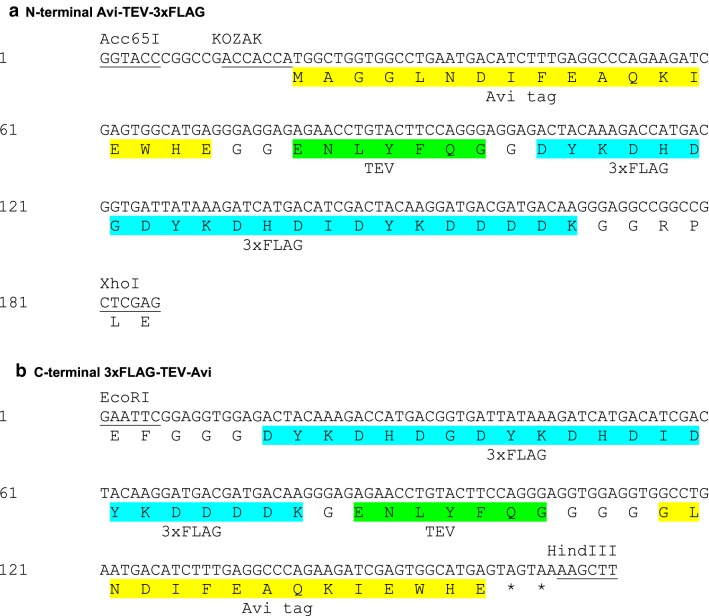
**a** Nucleotide sequence and translation of the N-terminal Avi-TEV-3xFLAG tandem affinity tag, cloned as an *Acc*65I/*Xho*I fragment in pBluescript SK (see also Additional file 1: Figure S1A). The Kozak sequence is underlined. **b** Nucleotide sequence and translation of the C-terminal 3xFLAG-TEV-Avi tandem affinity tag which was cloned as an *Eco*RI/*Hin*dIII fragment in pBluescript KS (see also Additional file 1: Figure S1B). Asterisks denote stop codons

**Fig. 2:**
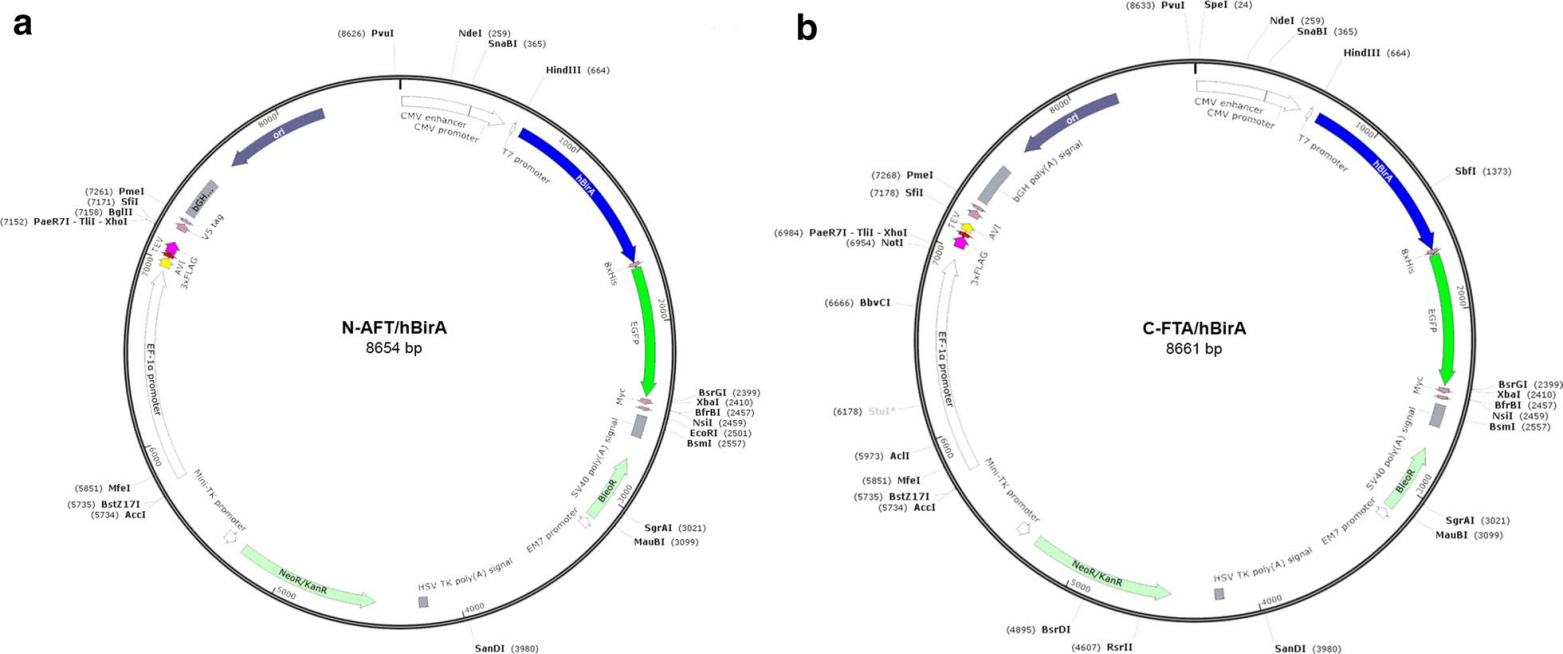
Maps of plasmids N-AFT/hBirA (**a**) and C-FTA/hBirA (**b**) showing the cloning of the N-terminal Avi-TEV-3xFLAG or of the C-terminal 3xFLAG-TEV-Avi tandem affinity tags under the EF1α promoter and of the hBirA-GFP under the CMV promoter of pBUDNeo. Unique restriction sites for cloning in-frame to the tags include *Xho*I, *Bgl*II and *Sfi*I in N-AFT/hBirA and *Not*I and *Xho*I in C-AFT/hBirA

Expression vectors were constructed using the mammalian expression vector pBudCE4.1 (Life Technologies) modified by the addition of the thymidine kinase-neomycin resistance gene (TK Neo^R^) gene to yield vector pBUDNeo. The mammalian codon-optimized (“humanized”) hBirA-GFP fusion [8, 9] was cloned in pBUDNeo downstream of the CMV promoter to generate hBirA-GFP pBUDNeo (Fig. 2). In parallel, the N-terminal Avi-TEV-3xFLAG (ATF) and C-terminal 3xFLAG-TEV-Avi (FTA) tandem affinity tag sequences (Fig. 1) were assembled by gene synthesis (GeneArt, Life Technologies), verified by sequencing and cloned in pBluescript SK (Agilent Technologies) (for ATF, Additional file 1: Figure S1A) or pBluescript KS (Agilent Technologies) (for FTA, Additional file 1: Figure S1B) to generate two general-purpose plasmids carrying the N- or C-terminal tandem affinity tagging sequences. Next, the N-terminal ATF or C-terminal FTA tagging sequences were cloned downstream of the EF1α promoter in plasmid hBirA-GFP pBUDNeo, to generate plasmids N-ATF/hBirA or C-FTA/hBirA (Fig. 2a). The GATA1 expression constructs were generated by in-frame cloning of the GATA1 cDNA to the N-terminal ATF/hBirA vector or the C-terminal FTA/hBirA vector. The GATA-1 fusions to the tags in the final expression plasmids were verified by sequencing. Further details regarding the construction of the plasmids described here are available upon request.

#### Transient transfections

HEK293 cells (60–70% confluency) were transiently transfected using the JetPEI™ DNA transfection reagent according to the manufacturer’s instructions (Source Bioscience, Nottingham, UK). 8–10 μg of plasmid DNA was used per 10 cm plate transfected.

#### Nuclear extracts

Transiently transfected cells were harvested after 24 h and nuclear extracts were made as previously described [10]. Nuclear proteins were quantitated using Bio-Rad’s colorimetric Protein Assay kit I.

#### Antibodies

Anti-GATA-1 N6 rat monoclonal antibody (sc-265, Santa Cruz Biotechnology); anti-GFP a mouse monoclonal antibody (sc-9996, Santa Cruz Biotechnology); anti-HA rabbit polyclonal antibody (sc-805, Santa Cruz Biotechnology); M2 FLAG mouse monoclonal antibody (Sigma Aldrich).

#### Other methods

Streptavidin pulldown, SDS-PAGE electrophoresis and Western immunoblotting were all done as described in [11]. Streptavidin–horseradish peroxidase (HRP) conjugate was purchased from Perkin Elmer.

### Results

We generated a series of constructs for the N- or C-terminal biotinylation tagging of proteins which include a triple (3x) FLAG tag fused in tandem to the Avi biotinylatable tag [12] allowing for the option of tandem affinity purification. The two tags are separated by a TEV protease cleavage site (Fig. 1). The N-terminal Avi-TEV-3xFLAG and the C-terminal 3xFLAG-TEV-Avi sequences were first cloned in pBluescript SK and KS, respectively (Additional file 1: Figure S1), thus generating two platform constructs that can be used for cloning any cDNA of interest in-frame to the N- or C-terminal tags, followed by re-cloning of the tagged sequences to an expression vector of choice or to a gene locus of interest, for example by CRISPR/Cas9 mediated approaches.

With the aim of generating a single construct for the expression of either the N-terminally or C-terminally tagged nuclear protein of interest and of the mammalian codon optimized hBirA biotin ligase, we used the pBudNeo expression vector which contains two independent transcription units driven by the elongation factor 1α (EF1α) and cytomegalovirus (CMV) promoters (Fig. 2) and which is well suited for stable or transient mammalian cell transfections. The N- or C-terminal tandem affinity tags were cloned under the control of the EF1α promoter using restriction sites that allow the in-frame cloning of cDNAs by PCR, whereas the hBirA-GFP fusion was cloned under the control of the CMV promoter (Fig. 2a, b). The hBirA biotin ligase-GFP fusion allows one to use GFP fluorescence to assess transfection efficiency and hBirA expression levels and to sort transfected cells from a pool of cells [9].

**Fig. 3:**
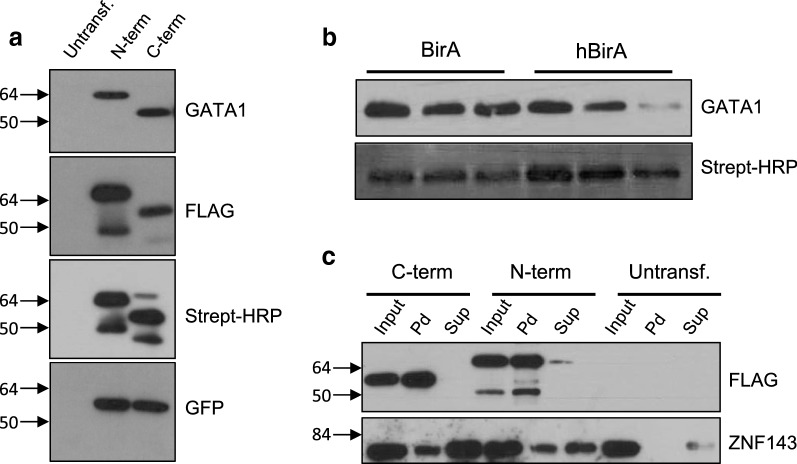
**a** Expression of transiently transfected N-terminally or C-terminally tandem affinity tagged GATA1 detected by anti-GATA-1 immunoblot (top panel), anti-FLAG immunoblot (second panel). Biotinylation of tagged GATA1 was detected by streptavidin–HRP (third panel), whereas hBirA-GFP expression was detected by anti-GFP immunoblot (last panel). The difference in mobility observed between the N-terminally and the C-terminally tagged GATA1 constructs is due to the presence of extra codons that were introduced during cloning of the GATA1 cDNA downstream of the N-terminal tag sequences. The extra bands detected in the anti-FLAG and streptavidin–HRP blots appear to be non-specific as they are not detected by anti-GATA1. **b** Detection of biotin-tagged GATA1 using anti-GATA1 antibody (upper panel) and streptavidin–HRP (lower panel) in dilutions of nuclear extracts from HEK293 cells transiently transfected with 3xFLAG-TEV-Avi-GATA1/BirA pBUDNeo (lanes labeled as BirA) or with 3xFLAG-TEV-Avi-GATA1/hBirA-GFP pBUDNeo (lanes labeled as hBirA). **c** Streptavidin pulldowns of nuclear extracts from HEK293 cells transiently transfected with N- or C-terminally tagged GATA-1 constructs. Top panel: detection with anti-FLAG antibody; lower panel: detection of the known GATA1 interacting protein partner ZNF143 co-precipitated with biotinylated GATA1 by streptavidin pulldown. Molecular weight markers (arrows) are in kilodaltons

In order to test these constructs, we cloned the murine GATA1 cDNA in-frame to the N- or C-terminal tags downstream of the EF1α promoter and transiently transfected them in HEK293 cells. GATA1 is an essential hematopoietic transcription factor which has been studied extensively through the application of biotinylation tagging [11, 13, 14]. Nuclear extracts were isolated at 24 h post-transfection and expression of hBirA-GFP was confirmed using an anti-GFP antibody (Fig. 3a). We next confirmed expression of N- or C-terminally tagged GATA1, as detected by anti-GATA1 and anti-FLAG antibodies, whereas biotinylation of tagged GATA1 was confirmed using streptavidin–HRP (Fig. 3a). We also tested the efficiency of biotinylation mediated by the mammalian codon optimized hBirA compared to the original bacterial BirA biotin ligase. To this end, we transiently transfected HEK293 cells with the pBUDNeo-based vector expressing the C-terminally tagged GATA1 together with hBirA-GFP (Fig. 2b) or with an identical vector expressing the *E. coli* 3xHA-tagged BirA instead of hBirA-GFP. We used dilutions of nuclear extracts normalized for GATA1 expression from the two transfections (with hBirA or *E. coli* BirA) to assay for biotinylation of tagged GATA1. From this it is clear that hBirA is more efficient in biotinylating tagged GATA1, since stronger signals using streptavidin–HRP were obtained throughout the hBirA nuclear extract dilutions compared to the BirA dilutions (Fig. 3b).

We also used streptavidin pulldowns in order to assess the biotinylation efficiency of the N- or C-terminally tagged GATA1 protein. In both cases, as detected by GATA1 antibody and streptavidin–HRP, we saw that almost all of the biotin-tagged GATA1 protein is bound and pulled down by streptavidin beads, indicating a very high efficiency of tagged GATA1 biotinylation in HEK293 cells (Fig. 3c). In addition, we also show that streptavidin pulldown of N-terminally or C-terminally tagged GATA1 results in the co-precipitation of the endogenous transcription factor ZNF143 which has been previously reported to interact with GATA1 [15, 16], thus demonstrating the utility of these constructs in investigating protein–protein interactions. Similar results were also obtained in immunoprecipitation experiments using an anti-FLAG antibody (data not shown).

### Discussion

We describe here the generation of expression vectors for the efficient biotinylation tagging of proteins in mammalian cells. Specifically, we generated two platform constructs bearing in tandem 3xFLAG and biotinylatable Avi tags for the N- or C-terminal tagging of target proteins of interest, which can then be re-cloned into mammalian expression vectors of choice. The presence of two affinity tags in tandem and of an intervening TEV protease cleavage site allows downstream tandem affinity purification of tagged proteins from nuclear extracts (for example, see [7]). We also generated mammalian expression vectors carrying on the same plasmid N- or C-terminal 3xFLAG and Avi tandem affinity tags under the control of the EF1α promoter and the mammalian codon optimized hBirA fused to GFP under the control of the CMV promoter. These vectors allow for transient or stable expression and biotinylation in mammalian cells of N- or C-terminally tagged proteins using a single plasmid as vector. All the above vectors provide utility and flexibility in affinity purification protocols employing in vivo metabolic biotinylation tagging and the advantages associated with it.

## Limitations

The expression vectors described here rely on their transient or stable transfection in cultured mammalian cells. As such, they are subject to the limitations of transfection assays such as low transfection efficiencies and low levels, or altogether absent, expression as a result of chromosomal position effects at the site of integration in stably transfected cells. Furthermore, expression levels of cDNAs cloned in the expression vectors described here cannot be in any way adjusted, as for example in inducible expression systems. This may result in situations where overexpression of a given cDNA cloned in the expression vectors described here may prove deleterious to the cells.

## Additional file

**Additional file 1: Figure S1.** Restriction maps of plasmid Avi-TEV-3xFLAG_pBS SK (A) and of plasmid 3xFLAG-TEV-Avi_pBS KS (B).

## Abbreviations

TEV: tobacco etch virus
GFP: green fluorescent protein
cDNA: complementary DNA
*K*d: dissociation constant
TK: thymidine kinase
Neo^R^: neomycin resistance
HRP: horseradish peroxidase
EF1α: elongation factor 1α
CMV: cytomegalovirus
