# Supplementary figures and images for: Mammalian expression vectors for metabolic biotinylation tandem affinity tagging by co-expression in cis of a mammalian codon-optimized BirA biotin ligase
Source: BMC Res Notes. 2018 Jun 14;11:390. doi: 10.1186/s13104-018-3500-9 (PMC6001059; doi:10.1186/s13104-018-3500-9)

A.

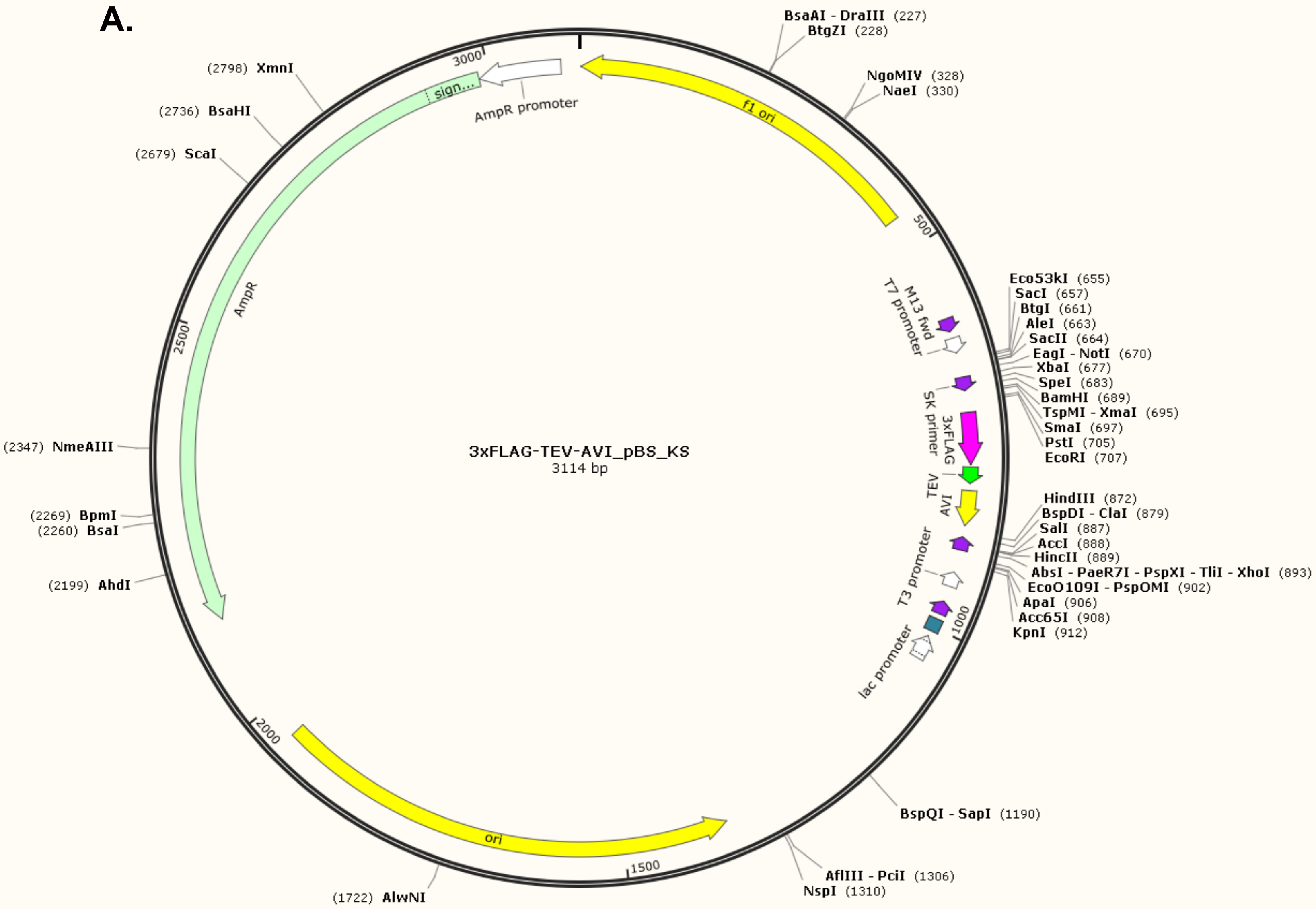

B.

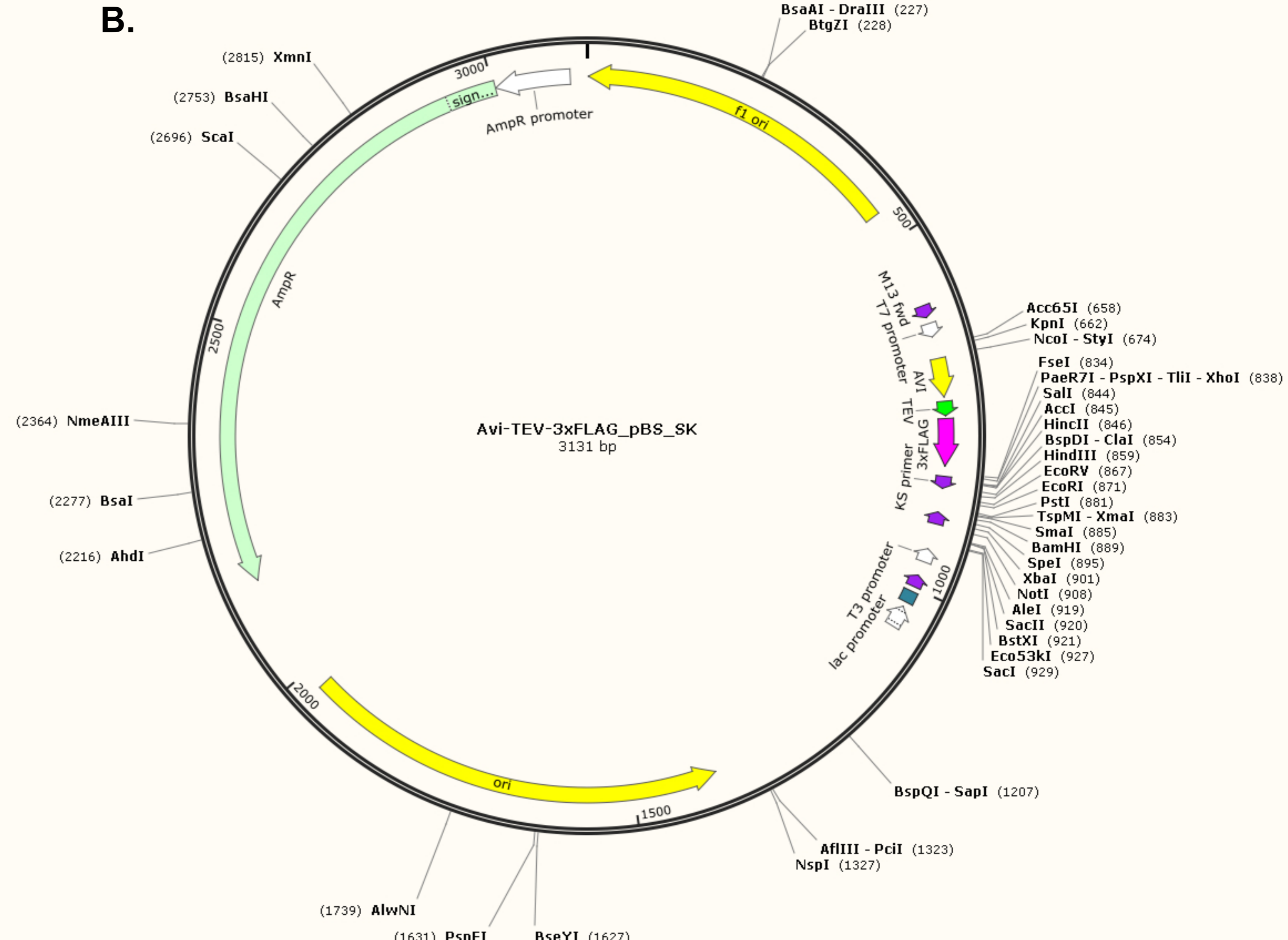

Supplement: Supplementary file 1 — Additional file 1: Figure S1. Restriction maps of plasmid Avi-TEV-3xFLAG_pBS SK (A) and of plasmid 3xFLAG-TEV-Avi_pBS KS (B). [file 13104_2018_3500_MOESM1_ESM.pdf]
